# Peri‐arterial pathways for clearance of α‐Synuclein and tau from the brain: Implications for the pathogenesis of dementias and for immunotherapy

**DOI:** 10.1002/dad2.12070

**Published:** 2020-07-28

**Authors:** Jacqui Nimmo, David A. Johnston, J. C. Dodart, Matthew T. MacGregor‐Sharp, Roy O. Weller, James A. R. Nicoll, Ajay Verma, Roxana O. Carare

**Affiliations:** ^1^ Faculty of Medicine University of Southampton Southampton UK; ^2^ United Neuroscience Dublin Republic of Ireland

**Keywords:** Immunotherapy, IPAD, perivascular drainage, tau, α‐synuclein

## Abstract

**Introduction:**

Accumulation of amyloid beta (Aβ), α‐synuclein (αSyn), and tau in dementias indicates their age‐related failure of elimination from the brain. Aβ is eliminated along basement membranes in walls of cerebral arterioles and leptomeningeal arteries (intramural peri‐arterial drainage [IPAD]); IPAD is impaired with age. We test the hypothesis that αSyn and tau are also eliminated from the normal brain along IPAD pathways.

**Methods:**

Soluble αSyn or tau was injected into mouse hippocampus. Animals were perfused 5 minutes to 7 days post‐injection. Blood vessels were identified by ROX‐SE for light‐sheet and immunolabeling for confocal microscopy. IPAD was quantified by measuring the proportion of arterioles with αSyn/tau.

**Results:**

αSyn and tau are eliminated from the brain by IPAD but with different dynamics.

**Discussion:**

Age‐related failure of IPAD may play a role in the pathogenesis of synucleinopathies and tauopathies. αSyn persists within IPAD at 24 hours, which may affect immunotherapy for αSyn.

## 1 INTRODUCTION

Many dementias are characterized by the accumulation of amyloid beta (Aβ), α‐synuclein (αSyn), tau, or other proteins in the brain, indicating an age‐related failure of elimination of these proteins. If preventative measures and therapeutic strategies are to be further developed for the treatment of dementias, a firm understanding is required of how soluble proteins are eliminated from the brain, why such elimination fails with age, and how elimination can be facilitated.

So far, most attention on pathways for elimination and why they fail has centered on Alzheimer's disease (AD). Plaques of insoluble Aβ are one of the hallmarks of AD but levels of soluble Aβ in the interstitial fluid (ISF) of the brain are also raised which suggests a failure of elimination of soluble Aβ and loss of homoeostasis of the extracellular environment in the brain.^1^, ^2^ Soluble Aβ in the brain is degraded by tissue enzymes such as neprilysin, absorbed into the blood, and is also eliminated from the brain, with ISF, along peri‐arterial lymphatic drainage pathways.^3^, ^4^ As there are no conventional lymphatics in the brain, ISF and soluble Aβ drain to lymph nodes with other solutes along basement membranes (BMs) in the walls of cerebral capillaries and arteries to cervical lymph nodes; this has been termed intramural peri‐arterial drainage (IPAD).^5^, ^6^, ^7^ Experimental studies in mice have shown that IPAD is impaired as arteries age, and such age‐related impairment in humans is associated with deposition of insoluble Aβ in IPAD pathways as cerebral amyloid angiopathy (CAA).^8^, ^9^, ^10^ In addition to the accumulation of Aβ in the brain, failure of IPAD appears to contribute to white matter hyperintensities in which fluid accumulates in the white matter in association with increasing age and CAA.^11^

As well as Aβ, insoluble and soluble αSyn accumulates in the brain in Parkinson disease and Lewy body dementias.^12^ Synucleins regulate the kinetics of synaptic vesicle endocytosis,^13^ and in the brains of patients with Parkinson disease and Lewy body diseases αSyn accumulates in neurons as insoluble Lewy bodies.^14^, ^15^ In normal human and mouse brains there is a level of 0.5 to 8.0 ng/mL of extracellular αSyn in the ISF, but this level is higher in mouse models of Parkinson disease,^16^ indicating a disease‐related failure of elimination of αSyn from the brain.

In the normal brain, tau is highly enriched in neurons in which it modulates microtubule dynamics and plays a role in mediating axonal transport and synaptic structure and functions together with neuronal signaling pathways.^17^ Although Tau accumulates within neurons as neurofibrillary tangles and in other cells in the brain in tauopathies and Lewy body dementias it is also present in the ISF and this level is found to increase in transgenic mice overexpressing tau compared to wild type mice^18^, ^19^

Mechanisms for intracellular degradation of αSyn and tau have been investigated extensively investigated.^20^, ^21^ It also appears that αSyn and tau are propagated between cells in the brain and this would involve αSyn and tau in the ISF in intercellular spaces in the brain.^16^, ^22^, ^23^, ^24^ Immunotherapies have been introduced for the removal of αSyn, tau, and Aβ from the brains of patients with dementia.^25^ Most research, however, has concentrated on immunotherapy for AD, entailing the removal of Aβ. Despite the successful removal of insoluble Aβ plaques from the cerebral cortex, immunotherapy for Aβ is associated with a rise in soluble Aβ in the ISF of the brain parenchyma and an increase in the severity of CAA.^26^, ^27^ Such complications suggest that impaired IPAD prevents the complete removal of Aβ from the brain and therefore limits the effectiveness of the immunotherapy. In effect, impaired IPAD is a rate‐limiting step in immunotherapy for AD.

To ensure maximum effectiveness of immunotherapy for αSyn and tau, it is crucial to understand the pathways by which these proteins in their soluble forms are eliminated from the brain. The major objective of this study is to determine whether soluble αSyn and tau drain from the brain along IPAD pathways and how the dynamics of this drainage compare with the elimination of soluble Aβ. Such knowledge would indicate whether therapies to improve the efficiency of IPAD in elderly individuals should be included in protocols for immunotherapy.

**RESEARCH IN CONTEXT**

1. Systematic review: The accumulation of α‐synuclein (αSyn), tau, and amyloid beta (Aβ) in the brain is a hallmark of dementias, indicating the failure of elimination of these proteins with advancing age. Soluble Aβ drains from the brain along basement membranes in the walls of arteries; such drainage is impaired with age and after immunization against Aβ. The dynamics for the drainage of αSyn and tau from the brain are unclear and are the focus of this article.
2. Interpretation: Results show that both αSyn and tau are eliminated from the brain along artery walls with dynamics that are similar, but not identical, to those of Aβ. Age‐related impairment of peri‐arterial drainage for αyn and tau is likely to be an important factor in the accumulation of these proteins in aging and demented brains.
3. Future directions: Therapeutic strategies to enhance peri‐arterial drainage from the aging brain will improve the elimination of αSyn, tau, and Aβ in the prevention of dementias, and these strategies should be considered also in the context of immunotherapy.

In this study we test the hypothesis that αSyn and tau are eliminated from the brain along IPAD pathways but possibly with dynamics that vary from those of Aβ. αSyn and tau were injected separately into the mouse hippocampus, and the distribution of these two proteins over space and time in the brain parenchyma and in the walls of blood vessels was recorded by light‐sheet and confocal microscopy at 5 minutes, 30 minutes, 24 hours, or 7 days post‐injection. The results allow the dynamics of IPAD for αSyn and tau to be assessed and to be compared with the IPAD dynamics for Aβ.

## 2 METHODS

### 2.1 Animals

Male C57BL/6 10 to 11‐week‐old mice were housed in groups of 5‐10, kept under a standard 12‐hour light/dark cycle, and fed a standard RM1 chow diet (SDS, UK) and water *ad libitum*. All procedures were carried out in accordance with animal care guidelines stipulated by the United Kingdom Animals (Scientific Procedures) Act 1986, Home Office license.

### 2.2 Intracerebral injections

#### 2.2.1 Anesthesia

To maintain a sufficient heart rate and oxygen saturation, 2% Isoflurane was mixed with concentrated O_2_ (1.7 L/min) and used throughout the procedure^28^ with a standard system of anesthesia. Internal body temperature was regulated at 37°C using a rectal probe, a homeothermic blanket, and a temperature control system (BASi).

#### 2.2.2 Labeling of tau protein

Recombinant human 2N4R‐tau (Abcam, ab87400) was prepared in sterile filtered phosphate‐buffered saline (PBS) (pH 8.7) using a 7K MWCO Zeba spin desalting column (Thermo scientific, 89889) following the manufacturer's instructions. The resulting tau was concentrated to 0.2 mg/mL using VivaSpin concentrator (10000 MWCO, VivaScience). The tau was added to Alexa‐Fluor‐488‐TFP‐ester (Thermo Scientific, A37570) and mixed for 1 hour on a Cleaver vortex and incubated at 4°C overnight. Unconjugated dye was removed using Zeba columns. Tau‐Alexa488 conjugates contained two moles of dye per mole protein.

#### 2.2.3 Intracerebral injections

Surgery was performed using a stereotaxic frame (KOPH instruments, Model 900) with a digital manipulator (World Precision Instruments). A burr hole was created in the skull using a Tech2000 Micromotor drill (RAM Products, INC) with 0.7 mm burr. One‐half microliter of either 60 µM recombinant human αSyn(1‐140)‐HiLyteFluor‐488 (AnaSpec) or Tau‐Alexa488 was injected into the right hippocampus (anterior‐posterior 2 mm; medial‐lateral 1.5 mm; dorsal‐ventral 1.7 mm) using a 33 gauge Hamilton needle (Essex Scientific Laboratory Supplies Ltd.) and Microinjection syringe pump (UMP3T‐1; World Precision Instruments) at a rate of 0.25 µL/minute (supplementary figure 1a) The syringe was left in situ for 2 minutes to prevent reflux. Mice were killed by intracardiac perfusion of fixative after a further 5 minutes, 30 minutes, 24 hours, or 1 week (n = 5 per group). Buprenorphine was administered for analgesia.

#### 2.2.4 Perfusion fixation

For confocal analysis, at the end of each time point mice were terminally anesthetized with pentobarbitone (200 mg/kg) and intracardially perfused with PBS (0.01 M) followed by 4% paraformaldehyde (PFA) (in 0.01 M PBS, pH 7.4) at a rate of 5 mL/minute. For light‐sheet analysis, mice were perfused at 5 minutes or 24 hours with PBS followed by 20 mL of 40 µM 5‐Carboxy‐X‐Rhodamine Succinimidyl Ester (ROXSE) (to visualize blood vessels) at 3 mL/min, and then 4% PFA at 5 mL/min. Brains were immersed in 4% PFA for 3 hours at 4°C and then cryoprotected in 30% sucrose.

### 2.3 Tissue processing for light‐sheet microscopy

Brains were dehydrated in 50% to 100% methanol concentrations (12 hours each at RT). Brains were delipidated in 30% dichloromethane (DCM) 60% methanol for 1 hour, followed by 100% DCM for 4 hours, and then cleared in Dibenzyl ether (DBE) for over 12 hours. Brains were imaged using light‐sheet microscope (Ultramicroscope II, LaVision Biotec).

### 2.4 Immunohistochemistry for confocal microscopy

Twenty micrometer coronal slices were prepared on a Leica Cryostat. Immunolabeling of α‐smooth‐muscle‐actin‐Cy3 (SMA) (1:200, Sigma‐Aldrich, Dorset, UK) and collagen IV (1:400, ABCAM, Cambridge, UK) was performed on sections 200 µm anterior to the injection site (supplementary Figure S1b). Immunolabeling of perivascular macrophages using CD206 (1:200, Serotec), which is uniquely expressed on perivascular macrophages^29^ or astrocytes (GFAP, 1:200, Dako), was performed on adjacent sections. Sections were scanned using an SP8 confocal‐laser scanning microscope (Milton Keys, UK).

### 2.5 Statistical analysis and quantification of αSyn‐positive blood vessels

The area selected for analysis encompassed the dentate gyrus extending to the CA1 region of the hippocampus proper. Blood vessels within the hippocampal sulcus were not quantified. The number of arterioles/venules/capillaries with αSyn or tau in their BMs was counted within this area and normalized to the total number of arterioles/venules/capillaries. Blood vessels were classified as capillaries (diameter <10 µm), arterioles (SMA‐positive blood vessels with diameter larger than 10 µm), or veins (SMA‐negative blood vessels with diameter larger than 10 µm).^3^, ^9^ One‐way analysis of variance was conducted with Bonferroni corrections for post hoc analysis.

## 3 RESULTS

### 3.1 Distribution of αSyn and tau

To establish the change in overall distribution of αSyn or tau tracers after their injection into the hippocampus, whole brains were imaged using light‐sheet microscopy at 5 minutes or 24 hours post‐injection (PI).

**FIGURE 1:**
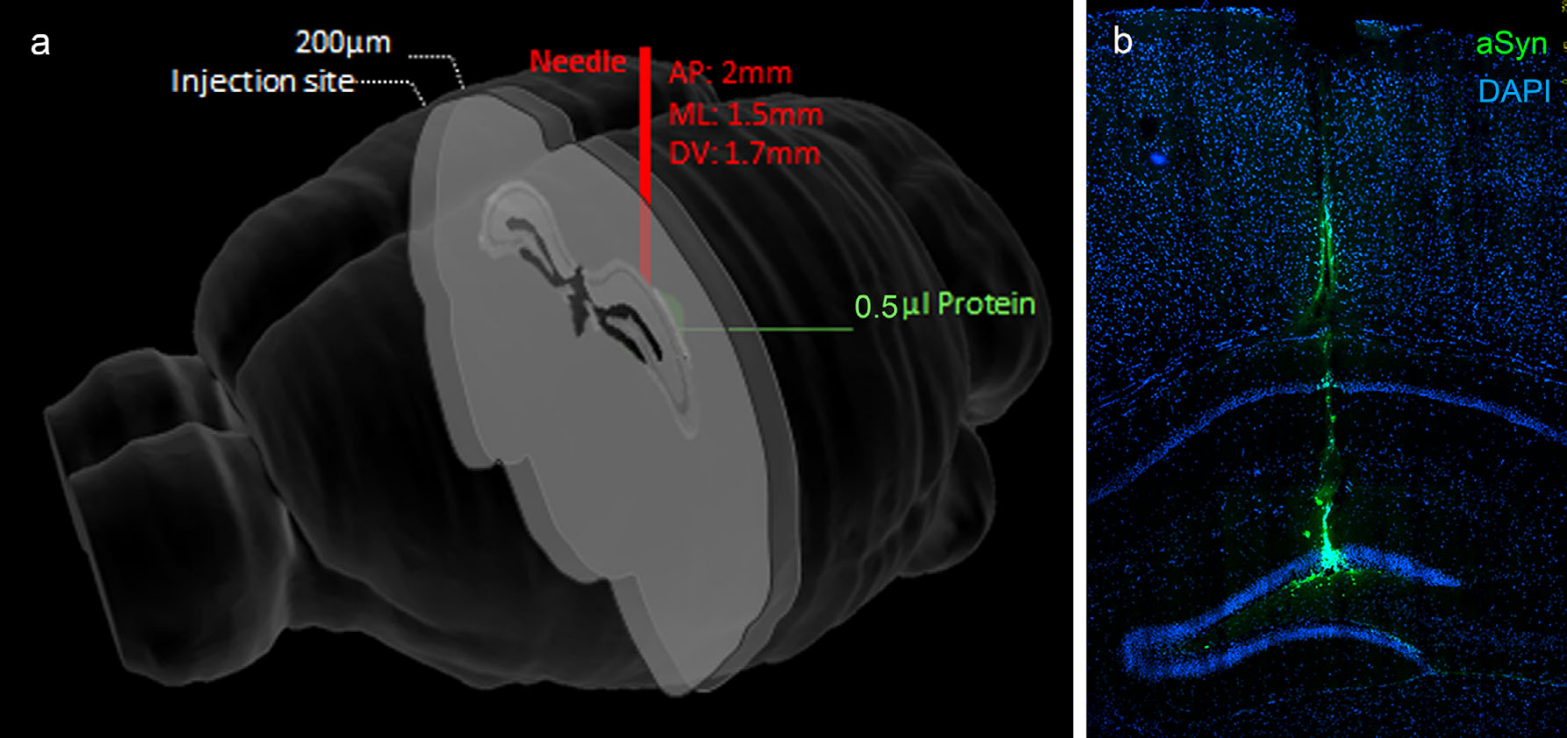
Site of injections of both α‐synuclein or tau into the mouse hippocampus. A, Diagrammatic representation of a mouse brain showing the site (in red) of 0.5 µL injections of either α‐synuclein or tau into the hippocampus. Brain sections for confocal microscopy and quantitative measurements were taken 200 µm posterior to the injection site. B, Site of injection of α‐synuclein (green). The needle track through the cortex is seen at the top of the figure leading to the injection site in the dentate gyrus of the hippocampus (DGH). No reflux was observed and no spread of α‐synuclein into the subarachnoid space, cerebral ventricles, or contralateral cerebral hemisphere was detected. A similar pattern of injection was achieved with tau. The section is stained with DAPI to show nuclei (blue) in the brain parenchyma.

Figure 1A is a diagram showing the site of injection of αSyn and tau, and Figure 1B illustrates the injection site of αSyn into the dentate gyrus of the mouse hippocampus.

**FIGURE 2:**
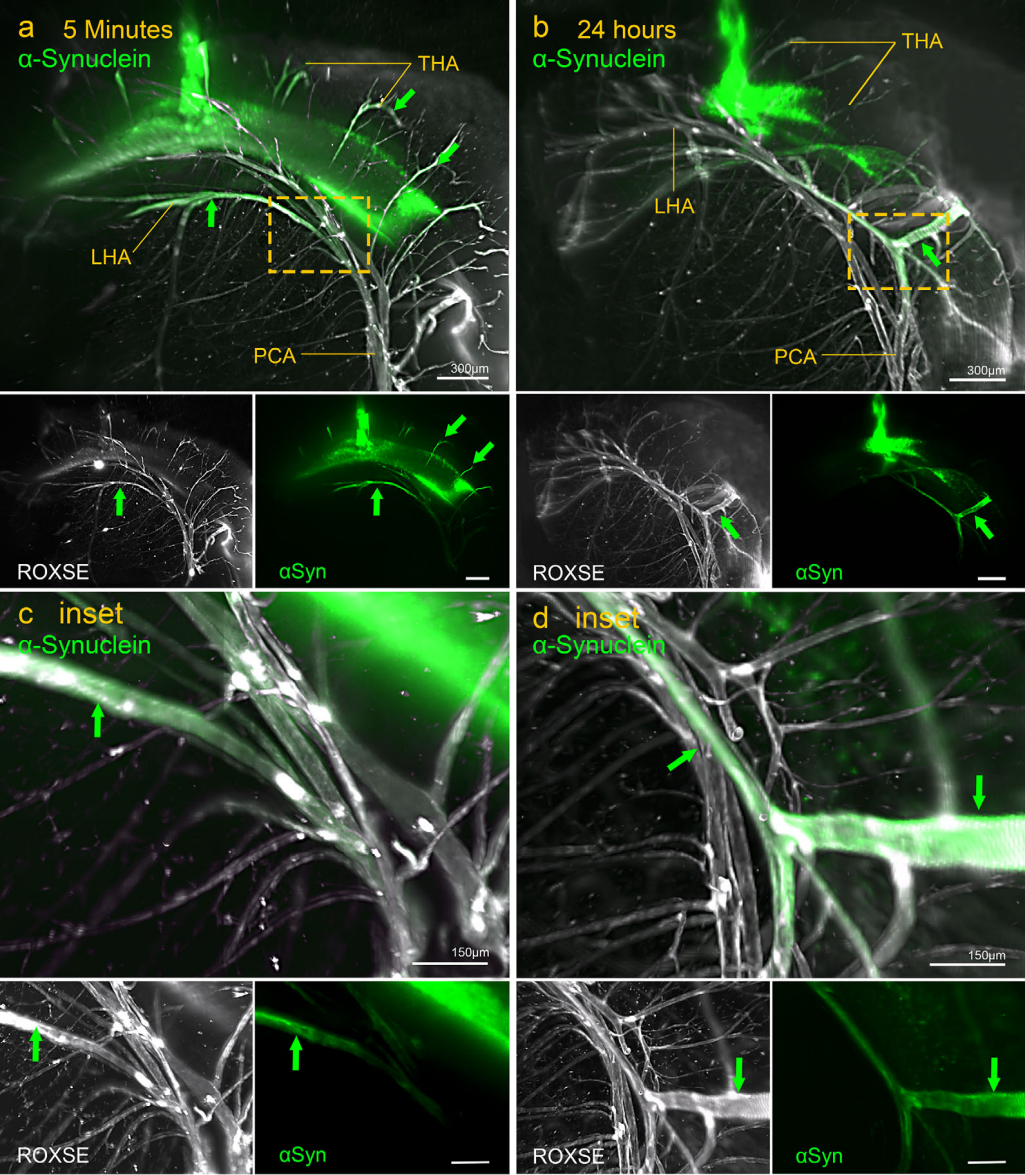
Light‐sheet images showing the distribution of α‐synuclein (αSyn) at 5 minutes (left‐hand images) and 24 hours (right‐hand images) post injection (PI). Green arrows indicate arteries with α‐synuclein in their walls. A, At 5 minutes PI, αSyn (green) is diffusely distributed within the dentate gyrus and in the walls of arteries (white) in the hippocampus. The transverse hippocampal arteries (THAs) and longitudinal hippocampal arteries (LHAs) that arise from the posterior cerebral artery (PCA) have αSyn in their walls (green arrows). Smaller images (below), separately stained white for blood vessels by ROXSE and green for αSyn show that branches of THAs (arrows) have αSyn in their walls. The boxed area in (A) is enlarged in (C) inset. B, At 24 hours PI, the diffuse αSyn in the dentate gyrus is notably reduced as is the αSyn surrounding the THAs and LHAs. Periarterial αSyn (green arrows) at 24 hours PI is more prominent in the walls of larger arteries like the PCA (see images separately stain for ROXSE and αSyn below). The boxed area is enlarged in (D) inset. Comparing 5 minutes, (C) inset, with 24 hours, (D) inset. Perivascular αSyn (green) is more prominent in the walls of larger arteries (green arrows) at 24 hours (D) inset than at 5 minutes (C) inset.

**FIGURE 3:**
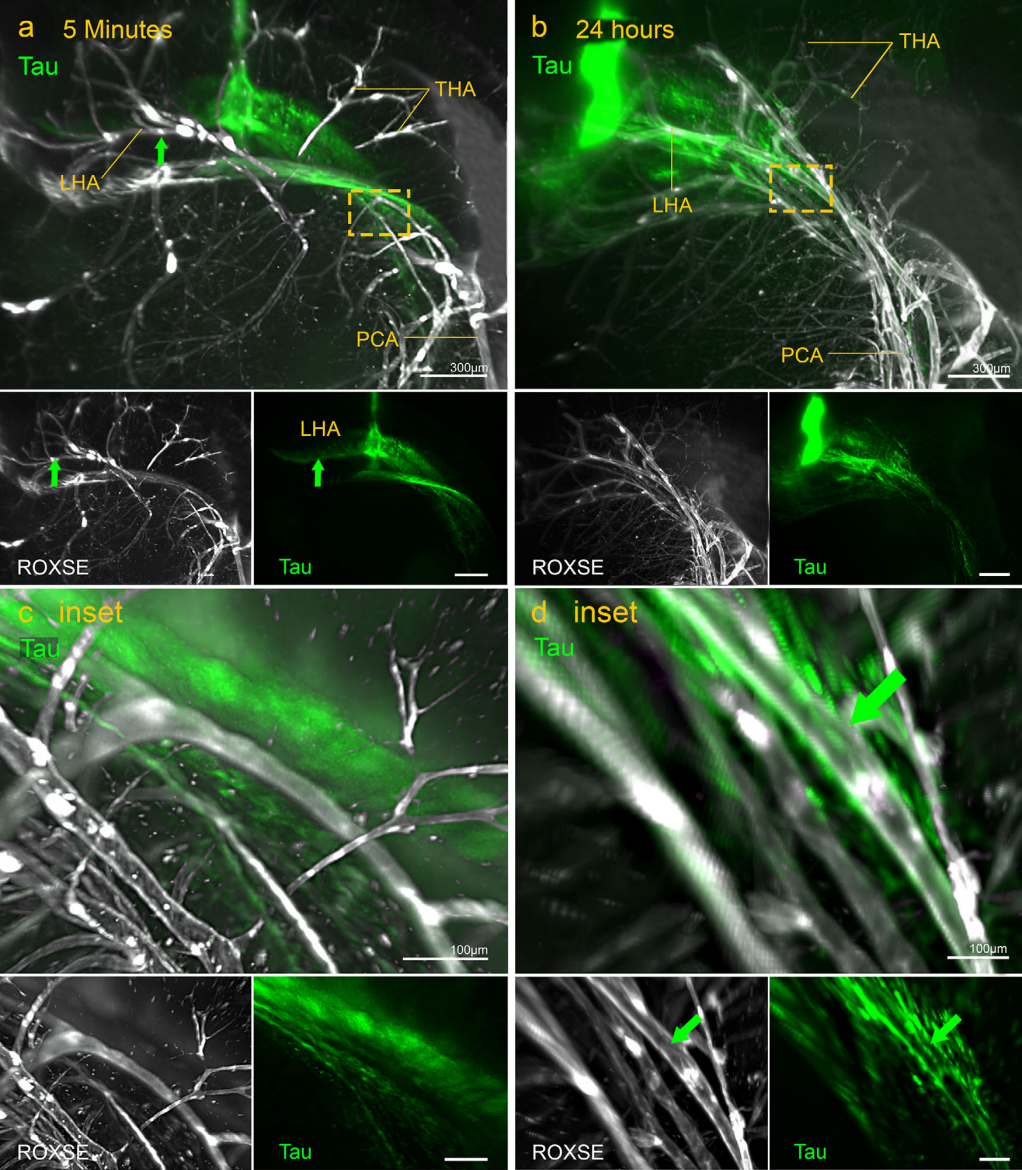
Light‐sheet images showing the distribution of tau (green) at 5 minutes (left‐hand images) and 24 hours (right‐hand images) post‐injection (PI). Green arrows indicate arteries with tau in their walls. A, tau (green) at 5 minutes PI is diffusely distributed within the dentate gyrus and in the walls of the hippocampal vasculature (white), in particular, branches of the longitudinal hippocampal arteries (LHAs) (see green arrows in (A) and in images stained for ROXSE and tau below). The boxed area is reproduced at higher magnification in (C) inset. B, At 24 hours PI, there is still some diffuse tau that is faintly detectable in the walls of the transverse hippocampal arteries (THA) or longitudinal hippocampal arteries (LHA), which branch from the PCA (see (D) inset). However, tau is present in oval profiles (see green arrow in (D) inset) that could represent perivascular macrophages (see Figure 6).

At 5 minutes PI, αSyn and tau diffused within the hippocampus (Figures 2A and 3A), and did not diffuse to surrounding brain regions even by 24 hours (Figures 2B and 3B). At 5 minutes PI, αSyn surrounded the transverse hippocampal arteries (THAs) and longitudinal hippocampal arteries (LHAs) (Figure 2A, Supplementary Movie 1), whereas minimal tau was localized to hippocampal blood vessels, mainly the posterior cerebral artery (PCA) (Figure 3A, Supplementary Movie 2). By 24 hours, diffuse αSyn had largely disappeared and perivascular αSyn was observed around the THAs and LHA toward the PCA (Figure 2C,D, Supplementary Movie 3). At 24 hours PI, tau was localized to more vessels and continued along branches of the PCA toward the surface of the brain (Figure 3B, Supplementary Movie 4). At 24 hours PI, tau appeared as small puncta in the parenchyma of the hippocampus as well as around blood vessels, contrasting with the diffuse appearance at 5 minutes PI (Figure 3C,D).

### 3.2 αSyn and tau enter IPAD

To investigate whether extracellular αSyn and tau enter IPAD pathways, the distribution of αSyn and tau was analyzed by confocal microscopy at 5 minutes, 30 minutes, 24 hours, and 7 days PI.

**FIGURE 4:**
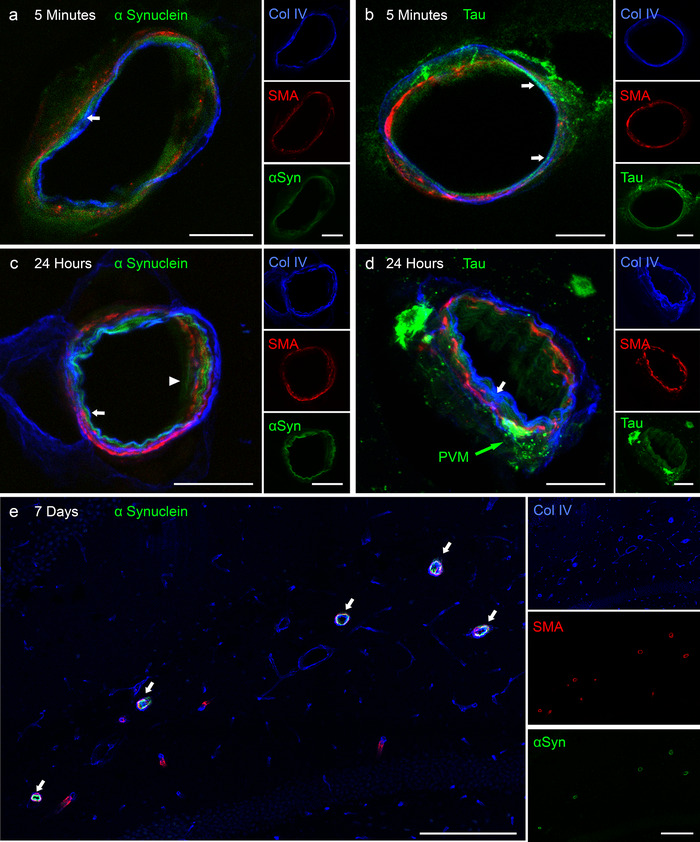
Distribution of α‐synuclein and tau within the walls of hippocampal arteries of adult mice. A, α‐synuclein (αSyn ‐ green) is diffusely distributed within the collagen IV of basement membranes (BMs ‐ blue) of arterioles at 5 minutes (arrow) and (C) at 24 hours (arrows). B, At 5 minutes PI, tau (green) is also diffusely distributed within collagen IV in BMs (blue) of arterioles (arrows). D, At 24 hours PI, very little tau (green) remains within the BMs of arterioles (arrow). Tau is also present in cells of the brain parenchyma surrounding the vessel. The cell closely applied to the vessel wall may be a perivascular macrophage (PVM). E, In contrast to tau, αSyn remains within BMs of arterioles in the superior hippocampal fissure (arrows) at 7 days. Co‐localization of αSyn with collagen IV can be seen as cyan regions in the arterioles (arrows). Characteristic pattern of IPAD is seen in (**c)** (arrowhead) as αSyn (green) spirals around smooth muscle cells (red) within the collagen IV basement membrane (blue). Scale bar = 10 µm

αSyn and tau were located in BMs of arterioles at 5 minutes PI, observed as regions of co‐localization between the green tracer and blue collagen IV producing a cyan color (Figure 4, arrows). At 24 hours PI, there were traces of tau in arteriolar BMs (Figure 4D); however, the majority appears intracellular, possibly within perivascular macrophages (Figure 6). αSyn persists in arteriolar BMs at 24 hours PI (Figure 4C) and 7 days, but only the leptomeningeal arteries of the superior hippocampal fissure contain αSyn (Figure 4E). Characteristic features of IPAD are observed in Figure 4D in which the αSyn was distinctly located within BMs surrounding smooth muscle cells, (Figure 4C, arrowhead).

**FIGURE 5:**
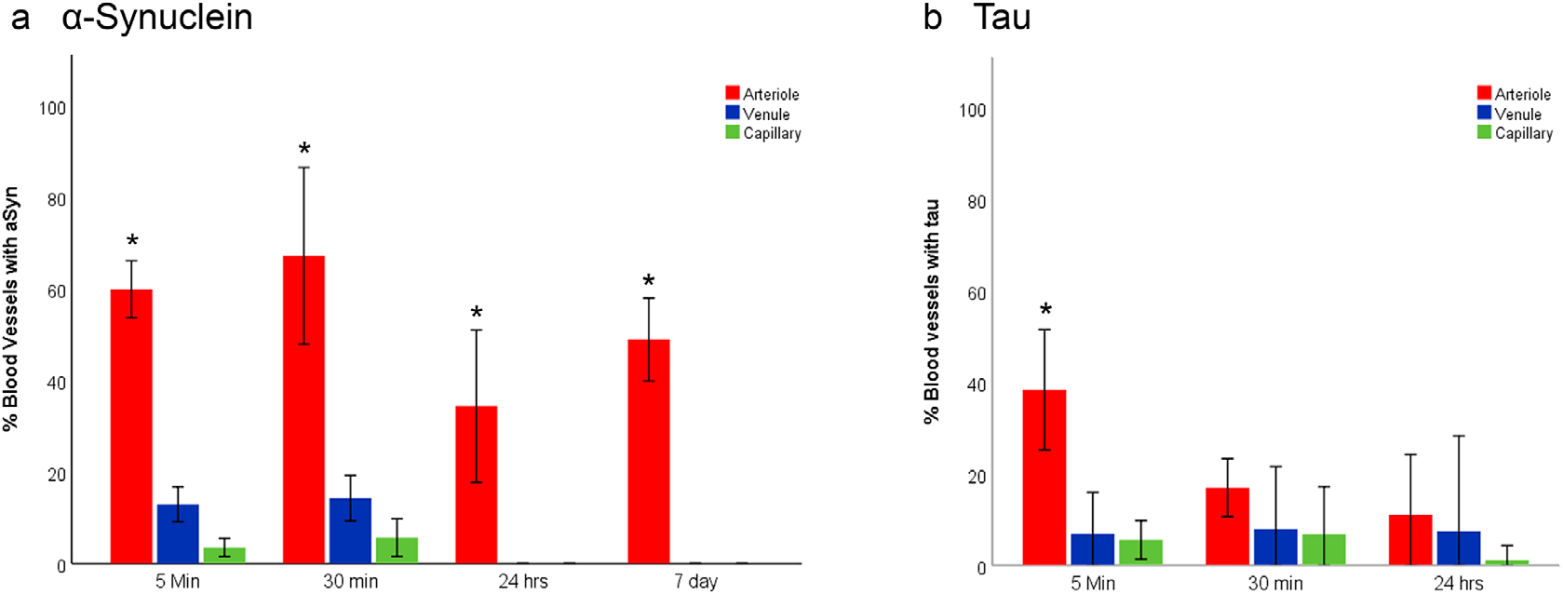
Quantification of the change in proportion of blood vessels containing α‐synuclein (A) or tau (B) over time. A), There is a significant reduction in the proportion of arterioles, venules, and capillaries containing α‐synuclein or tau at 24 hours. In the first 24 hours, there is a 25% to 33% decrease in the number of arterioles with α‐Synuclein within their walls. This decreased significantly to 20% to 40% by 24 hours (*P* < .014). The proportion of venules and capillaries containing α‐synuclein was significantly lower than arterioles at all time points (* = *P* < .0001). By 24 hours there were no venules or capillaries with αSyn in their basement membranes (BMs) (*P* ≤ .001). B, At 5 minutes PI of tau, there is a significantly higher proportion of arterioles (31% higher) compared to venules and capillaries with tau in their BMs (* = *P* < .0001). The proportion of arterioles with tau in their walls decreases significantly by 21% in the first 30 minutes PI (*P* = 0.01). This decreases significantly by a further 6% by 24 hours (*P* = 0.002). There is no difference in the proportion of venules and capillaries with tau in their walls over 24 hours. (one‐way ANOVA with Bonferroni corrections for multiple comparisons). Error bars represent ± 2SD

Quantification of the proportion of each blood vessel type that contained αSyn or tau over time is shown in Figure 5. The majority of vessels with tracer in their BMs were arterioles (5 minutes: αSyn: 60%; Tau: 40%), with <15% of venular and capillary BMs containing tracer at each time point. By 24 hours, αSyn‐ or tau‐positive venules and capillaries decreased to 0% and 6%, respectively. No difference between the proportion of arterioles, venules, or capillaries with tau in their walls was found between 30 minutes and 24 hours PI (Figure 5B). There was a significant decline in the proportion of αSyn‐positive vessels over 7 days, with the highest proportion occurring between 5 and 30 minutes (Figure 5A). Although there is a slight increase in αSyn‐positive arterioles at 7 days compared to 24 hours, this is not significant.

To confirm that the tracer was in the BMs of vessels and not within abutting astrocyte end feet, brain sections were immunolabeled with GFAP (Supplementary Figure S1). Slight co‐localization of perivascular GFAP with αSyn was observed at 30 minutes but not at 5 minutes or 24 hours. Tau was occasionally taken up by perivascular astrocytes at 5 minutes PI, but not at 30 minutes or 24 hours (Supplementary Figure S1b, d, f).

### 3.3 Tau, but not αSyn, is taken up by perivascular macrophages (PVMs)

**FIGURE 6:**
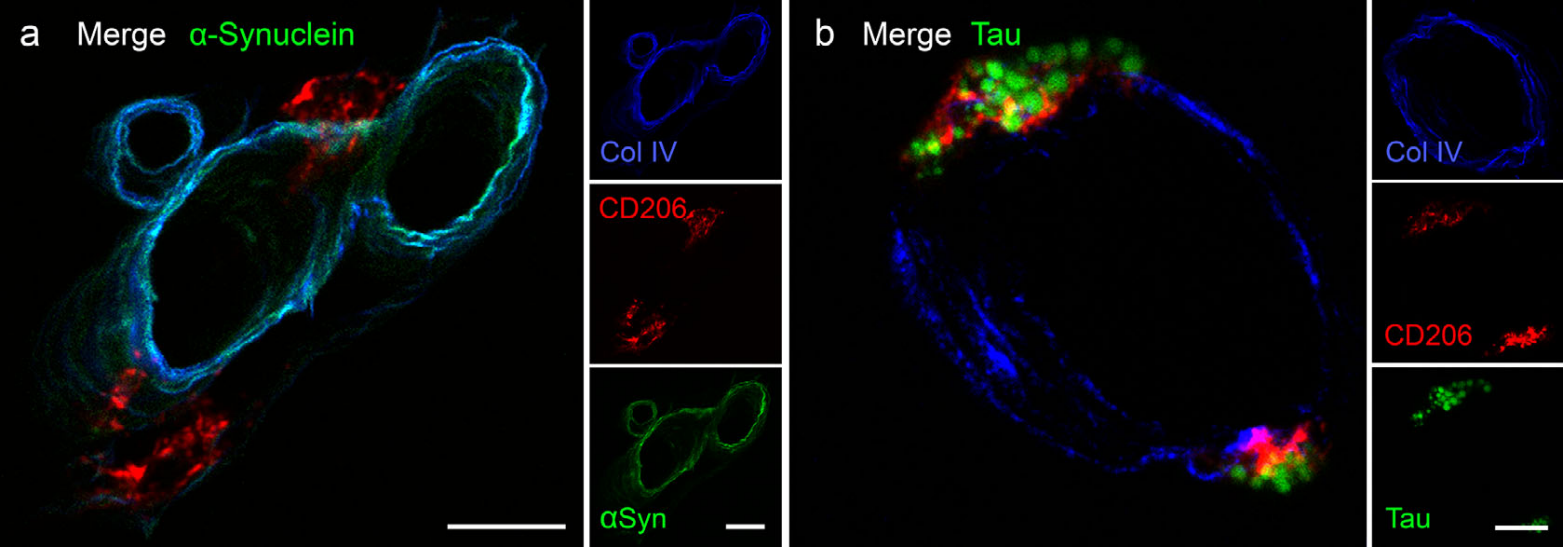
Perivascular macrophages (PVMs) are involved in the clearance of tau but not of α‐synuclein at 24 hours following hippocampal injection in 10‐week‐old mice. A, No co‐localization is seen between CD206‐positive PVMs (red) and α‐synuclein (green) at 24 hours PI or any other time point (data not shown). B, At 24 hours PI of tau, CD206‐positive PVMs (red) have engulfed punctate aggregates of tau (green). Scale bar = 10 µm

At 24 hours PI, perivascular tau is mainly within cells rather than in BMs (Figure 4D). To establish the perivascular location of tau at 24 hours, brain sections were immunolabeled with the PVM marker, CD206. αSyn did not co‐localize with CD206 at 24 hours (Figure 6A), or at any other time point. Tau, however, was found within CD206‐positive PVM at 24 hours (Figure 6B), but not at 5 to 30 minutes. The tau‐positive perivascular macrophages (PVMs) were mainly surrounding cortical arterioles, leptomeningeal arteries, a few veins, and some capillaries.

## 4 DISCUSSION

### 4.1 α‐Synuclein and tau are eliminated from the normal brain along periarterial pathways (IPAD) but with different dynamics

In the present study, light‐sheet microscopy and confocal microscopy have shown that αSyn and tau are eliminated from the brain along intramural peri‐arterial drainage (or IPAD) pathways in the walls of cerebral arterioles in a manner similar to the elimination of Aβ and other solutes.^3^, ^9^ This has important implications for the pathogenesis and management of dementias in which αSyn and tau accumulate in the brain.

There are some differences in the dynamics of IPAD between Aβ, αSyn, and tau. Examination of the drainage pattern of tau and αSyn through the whole hippocampus by light sheet microscopy showed that tau is not present within as many blood vessel walls as αSyn or Aβ. The number of arterioles with tau in their BMs (IPAD pathways) at 5 minutes PI is less than half of what has been reported for Aβ,^9^ suggesting that tau is not as readily cleared by IPAD as Aβ or αSyn. However, when tau enters IPAD pathways it exhibits dynamics similar to those of Aβ.^3^, ^9^ In young mice, both Aβ and tau enter IPAD pathways in the walls of arterioles within 5 minutes of injection into the brain, and by 24 hours, most of the Aβ and tau have disappeared from the drainage pathways but remain in perivascular macrophages. αSyn exhibits slightly different dynamics for IPAD; at 5 minutes after injection, αSyn is in IPAD pathways of hippocampal arterioles but in contrast to Aβ and tau, αSyn is still present and easily detectable in IPAD pathways at 24 hours and 7 days PI. It is unlikely that this is due to αSyn becoming “stuck” within the arteriole walls, as light‐sheet imaging clearly showed that αSyn moved away from the THAs and LHAs toward the PCA over time (Figure 2). αSyn does not appear to be taken up by PVMs as is the case with tau and Aβ.^30^ This may be due to the mechanism of uptake in which tau is more readily taken up by macropinocytosis,^31^ but internalization of αSyn has been found to occur in a receptor‐dependent mechanism.^32^, ^33^ The differences in dynamics between αSyn and tau may be due to interactions between αSyn and BMs in the IPAD pathways or, in the present experiments, it may be due to aggregates forming in the αSyn solution injected into the mice as shown in supplementary Figure S2. Previous studies have shown that particulate nanospheres of diameters >15 nm do not enter IPAD pathways but remain within the brain parenchyma,^34^ suggesting that larger aggregates may not drain as efficiently as soluble monomers. In addition to arterioles, αSyn and tau were located around capillaries and veins. Capillary walls are the initial pathways for IPAD, but there is no firm evidence that veins are involved in perivascular drainage of solutes from the brain. Some studies have suggested that veins are pathways for elimination of solutes from the brain into the lymphatic system.^35^ There are, however, major problems with these studies: (1) brains were examined 1 hour after the injection of tau or Aβ, so the time of maximum drainage along IPAD at 5 to 30 minutes had passed^3^; (2) IPAD appears to be driven by waves of smooth muscle cell contraction in the walls of arteries (vasomotion)^36^; (3) veins have few if any smooth muscle cells in their walls, so the motive force for perivenous drainage appears to be absent, whereas there is a continuous route along the walls of arteries in the brain to cervical lymph nodes no such route along veins has been characterized.^37^ In CAA, insoluble Aβ is deposited in IPAD pathways in the walls of cerebral arteries, whereas only minimal amounts of Aβ are associated with veins.^6^ It seems, therefore, that IPAD is the major route for drainage of ISF, Aβ, αSyn, tau, and other solutes from the brain to cervical lymph nodes and that there is no firm evidence for a perivenous route of drainage.

Impairment of IPAD associated with aging of cerebral arteries also plays a major role in the accumulation of Aβ in the brain in AD and in the deposition of Aβ in the IPAD pathways in CAA.^9^ CAA with the accumulation of insoluble Aβ and other proteins in IPAD pathways is major evidence that IPAD is involved in the drainage of solutes from the brain in humans.^9^, ^38^ The presence of tau in the walls of arteries in human brains has been reported,^39^ but tau does not accumulate as CAA in the same way as Aβ. Although Aβ shows a firm propensity to form fibrillary amyloid in IPAD pathways in CAA, tau does not appear to exhibit such properties of aggregation. There are no reports of αSyn in the walls of arteries in the human brain; however, it may be that angiopathy occurs only when a threshold of concentration of αSyn is reached. The results of the present study suggest that IPAD is a route for elimination of soluble αSyn from the brain, and therefore any intervention that may increase αSyn in the extracellular spaces may overcome the capacity of the clearance pathways for αSyn and may result in disruption of homeostasis and possibly also angiopathy, with other proteins building up as the normal capacity for IPAD is impaired.

### 4.2 Facilitating removal of α‐synuclein, tau, and Aβ by IPAD in the prevention and treatment of dementias and as an adjunct to immunotherapy

The finding that αSyn and tau drain from the brain along IPAD pathways in a manner similar to Aβ has implications for both treatment and immunotherapy for αSyn‐ and tau‐related dementias. Clinical trials to identify drugs that will facilitate the drainage of Aβ along the impaired IPAD pathways by stimulating the vascular smooth muscle cells as the motive force for IPAD are underway.^40^, ^41^ Immunization trials for Aβ are showing promise as reported very recently, with reductions in amyloid, phospho‐tau, and neurofilament light.^42^

Anti‐tau immunotherapy attempts are ongoing^43^ and two more recent active immunization trials are demonstrating modest improvements in neurodegeneration.^44^ Although the exact mechanisms for the action of the vaccines are not known, there is evidence for the uptake of tau by glial cells.^45^ Our present work shows the involvement in perivascular macrophages in the clearance of tau. Tau oligomeric specific monoclonal antibodies clear the brain of synuclein deposits as well as toxic forms of oligomeric synuclein,^46^ but the mechanism requires clarification.

Different immunotherapy compounds against αSyn are under development^43^, ^47^, ^48^ and one aspect to consider is that if immune complexes do form as in anti‐Aβ immunotherapies, in high doses they may block the IPAD pathways, as we show here that αSyn drains along the BMs of arterioles and remains there for 24 hours after its intraparenchymal injection.^49^, ^50^ Our results support the importance of IPAD for the clearance of aSyn and tau from the brain, and this should be considered in the context of immunotherapy trials against αSyn or tau.

## FUNDING INFORMATION

This work was supported by the Medical Research Council [MR/R502261/1] and United Neuroscience [517698101]. The funding agency did not play any active role in the scientific investigation and reporting of the study.

## DECLARATION OF INTERESTS

The authors have no competing interests to declare.

## Supporting information


**Supplementary Figure S1**: Astrocytes surrounding blood vessels after injection of α‐synuclein (αSyn) (**a, c, e**) or tau (**b, d, f**). (**a, c, e**) No αSyn staining (green) is associated with juxta‐vascular GFAP‐positive astrocytes (red) after injection of αSyn into the hippocampus of 10‐week‐old mice at **(a)** 5 minutes, **(c)** 30 minutes, or **(e)** 24 hours. A small degree of co‐localization of GFAP and αSyn is observed at 30 minutes **(c)** (arrows). Tau (green) co‐localized with GFAP‐positive astrocytes (red) at 5 minutes PI **(b)** but not at 30 minutes **(d)**, or 24 hours **(f)**. Scale bar = 10 µm

**Supplementary Figure s2**: Transition electron micrograph of **(a)** α‐synuclein **(b)** 2N4R tau used for injections. Light areas represent protein oligomers and larger aggregates. Monomers cannot be seen at this magnification and appear as a patchy background.

**Supplementary Movie 1**: 360^o^ rotation of light‐sheet imaging of the distribution of αSyn at 5 minutes after injection. Vasculature was visualized by intracardial perfusion of ROXSE (white). αSyn (green) is found diffusely distributed within the dentate gyrus and surrounding the hippocampal vasculature.

**Supplementary Movie 2**: 360^o^ rotation of light‐sheet imaging of the distribution of tau at 5 minutes after injection. Vasculature was visualized by intracardial perfusion of ROXSE (white). Tau (green) is found diffusely distributed within the dentate gyrus and surrounding the posterior cerebral artery.

**Supplementary Movie 3**: 360^o^ rotation of light‐sheet imaging of the distribution of αSyn at 24 hours after injection. Vasculature was visualized by intracardial perfusion of ROXSE (white). The perivascular αSyn (green) can be observed mainly within the posterior cerebral artery.

**Supplementary Movie 4**: 360^o^ rotation of light‐sheet imaging of the distribution of tau at 24 hours after injection. Vasculature was visualized by intracardial perfusion of ROXSE (white). Tau (green) accumulates around the transverse hippocampal arteries and longitudinal hippocampal arteries, which branch from the PCA.
