# Supplementary figures and images for: Peri‐arterial pathways for clearance of α‐Synuclein and tau from the brain: Implications for the pathogenesis of dementias and for immunotherapy
Source: Alzheimers Dement (Amst). 2020 Jul 28;12(1):e12070. doi: 10.1002/dad2.12070 (PMC7409108; doi:10.1002/dad2.12070)

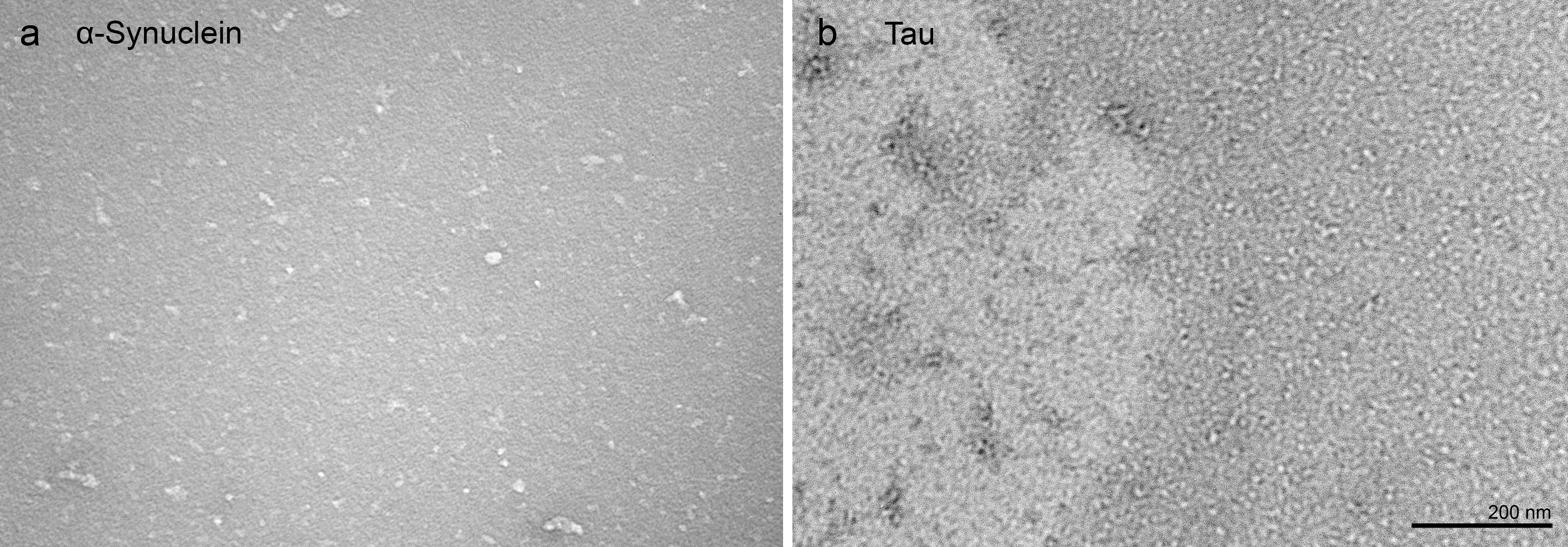

Supplement: Supplementary file 2 — Supplementary Figure s2: Transition electron micrograph of (a) α‐synuclein (b) 2N4R tau used for injections. Light areas represent protein oligomers and larger aggregates. Monomers cannot be seen at this magnification and appear as a patchy background. [file DAD2-12-e12070-s002.tif]
